# Using inflammatory index to distinguish asthma, asthma-COPD overlap and COPD: A retrospective observational study

**DOI:** 10.3389/fmed.2022.1045503

**Published:** 2022-11-17

**Authors:** Haiman Ma, Liu Yang, Lingli Liu, Ying Zhou, Xiaoya Guo, Shuo Wu, Xiaoxiao Zhang, Xi Xu, Xinyu Ti, Shuoyao Qu

**Affiliations:** ^1^Department of Pulmonary and Critical Care Medicine, Xijing Hospital, Air Force Medical University, Xi’an, China; ^2^Department of Clinical Laboratory, Xijing Hospital, Air Force Medical University, Xi’an, China

**Keywords:** asthma, asthma-COPD overlap, chronic obstructive pulmonary disease, inflammatory index, biomarker, routine blood test, pulmonary function

## Abstract

**Background:**

Although asthma and chronic obstructive pulmonary disease (COPD) are two well-defined and distinct diseases, some patients present combined clinical features of both asthma and COPD, particularly in smokers and the elderly, a condition termed as asthma-COPD overlap (ACO). However, the definition of ACO is yet to be established and clinical guidelines to identify and manage ACO remain controversial. Therefore, in this study, inflammatory biomarkers were established to distinguish asthma, ACO, and COPD, and their relationship with the severity of patients’ symptoms and pulmonary function were explored.

**Materials and methods:**

A total of 178 patients, diagnosed with asthma (*n* = 38), ACO (*n* = 44), and COPD (*n* = 96) between January 2021 to June 2022, were enrolled in this study. The patients’ pulmonary function was examined and routine blood samples were taken for the analysis of inflammatory indexes. Logistic regression analysis was used to establish inflammatory biomarkers for distinguishing asthma, ACO, and COPD; linear regression analysis was used to analyze the relationship between inflammatory indexes and symptom severity and pulmonary function.

**Result:**

The results showed that, compared with ACO, the higher the indexes of platelet, neutrophil-lymphocyte ratio (NLR) and eosinophil-basophil ratio (EBR), the more likely the possibility of asthma and COPD in patients, while the higher the eosinophils, the less likely the possibility of asthma and COPD. Hemoglobin and lymphocyte-monocyte ratio (LMR) were negatively correlated with the severity of patients’ symptoms, while platelet-lymphocyte ratio (PLR) was negatively correlated with forced expiratory volume in the 1 s/forced vital capacity (FEV_1_/FVC) and FEV_1_ percent predicted (% pred), and EBR was positively correlated with FEV_1%_ pred.

**Conclusion:**

Inflammatory indexes are biomarkers for distinguishing asthma, ACO, and COPD, which are of clinical significance in therapeutic strategies and prognosis evaluation.

## Introduction

Asthma and chronic obstructive pulmonary disease (COPD) are heterogeneous diseases characterized by chronic airway and/or lung diseases. Chronic respiratory diseases (CRD) were the third leading cause of death in the world, of which COPD was the most common cause of death, followed by asthma, and the number of deaths from COPD was eight times that of asthma (1, 2). Although the definitions of asthma and COPD are very clear (asthma is characterized by variable expiratory airflow limitation, and COPD is characterized by persistent airflow limitation), the definitions of the both are not mutually exclusive, which means that their diagnostic criteria may overlap (3). Therefore, it is difficult to completely distinguish asthma from COPD.

The characteristics of asthma and COPD can coexist in a given patient, who are diagnosed with asthma-COPD overlap (ACO), especially in smokers and the elderly (4). However, so far, there are few studies on ACO because it is excluded from most clinical studies on COPD and asthma (5, 6). Moreover, there is no universally recognized specific definition of ACO (4). In epidemiological investigations, the reported prevalence of ACO ranged from 9 to 55% of those with either diagnosis, which reflects the different diagnostic criteria used by different researchers (5). A large number of studies have found that the prognosis of patients with ACO is worse than those with asthma or COPD alone (7). In summary, because the diagnostic criteria of asthma and COPD may overlap, and the definition of ACO is ambiguous, clinicians need to subjectively combine the clinical characteristics of patients in addition to the objective indicators (e.g., computed tomography and pulmonary function test) for diagnosis (4). Therefore, it is urgent to find objective biomarkers to understand the pathological characteristics of asthma, ACO, and COPD, so as to assist clinical differential diagnosis, guide clinical treatment and evaluate prognosis (8).

Asthma and COPD are two common CRD of chronic airway inflammatory (9). Asthma is predominantly airway reactive inflammation mediated by T-helper (Th) two cells and type 2 innate lymphoid cells (ILC2), while COPD is considered a Th1-mediated inflammatory process (4). Neutrophil infiltration becomes evident in severe cases of asthma and COPD (10). Some of these complex inflammatory mechanisms may be common to both asthma and COPD, which means that clinical features of asthma and COPD may overlap (11). For example, eosinophil is not only the characteristic diagnostic biomarker for asthma (12), but also closely related to COPD exacerbations (13); neutrophil-lymphocyte ratio (NLR) is associated with the exacerbation of asthma and COPD (14–16). Although the pathogenesis of ACO is not well established (17), some scholars have proposed that systemic inflammation may be a contributing factor to the increased morbidity associated with the ACO (18). Meanwhile, it was found that ACO was a heterogeneous disease, and the classical diagnostic categories could not fully explain the great complexity of the potential inflammatory process that ultimately determined the treatment response (19–21). Consequently, it is in great clinical interest to analyze the inflammatory index in hemogram for establishing biomarkers of asthma, ACO, and COPD.

The purposes of this study are: (1) to compare the inflammatory indexes of asthma, ACO, and COPD, in order to understand their pathological characteristics; (2) to explore biomarkers that can distinguish three groups of CRD, in order to assist clinical differential diagnosis; and (3) to analyze the relationship between inflammatory indexes and the severity of patients’ symptoms and pulmonary function, in order to guide clinical treatment and evaluate the prognosis of the disease.

## Materials and methods

### Study design

This study was a single-center, retrospective observational study. In this study, participants diagnosed with asthma (22), ACO (22), and COPD (23) were included according to the diagnosis of clinicians. The participants’ baseline data were collected, their condition was evaluated, and pulmonary function was assessed. Blood samples were taken for complete blood counts (CBC) analysis in order to form new inflammatory indexes through hemogram combination. The levels of inflammatory indexes were compared among the three groups, the biomarkers that could distinguish the three groups of diseases were explored, and the relationship between inflammatory indexes and symptom severity and pulmonary function was analyzed.

### Setting and participants

In this study, patients with stable CRD diagnosed for the first time were recruited from Xijing Hospital of Air Force Medical University from January 2021 to June 2022. They were divided into asthma, ACO, and COPD according to physician diagnosis and spirometry. Individuals were excluded if they: (1) were hospitalized; (2) had used systemic corticosteroids and/or antibiotics in the past month; (3) had pneumonia, tuberculosis, and other respiratory diseases; (4) had malignant tumors, autoimmune diseases, systemic infectious diseases, and important organ dysfunction; and (5) had other reasons that are not suitable for the study.

### Measurements

Pulmonary function test (PFT): PFT was conducted with spirometer (MasterScreen PFT system, Jaeger, Hoechberg, Germany) following the American Thoracic Society/European Respiratory Society (ATS/ERS) standards (24). Participants also inhaled 400 μg Salbutamol for bronchodilation test.

Routine blood test: 2–5 ml of peripheral venous blood was drawn into ethylenediamine tetraacetic acid (EDTA) anticoagulant blood tubes according to the standard procedure of venipuncture (25). All samples were analyzed for CBC.

### Statistical analysis

The software SPSS 26.0 (IBM Corp., Armonk, NY, USA) was used for statistical analysis. The results are described as median interquartile range (IQR). The differences between more than two groups were analyzed by Kruskal–Wallis test according to the distribution of data points. Logistic regression analysis was used to predict the biomarkers that could distinguish the three diseases. Linear regression analysis was used to analyze the relationship between inflammatory indexes and symptom severity and pulmonary function. Statistical significance was accepted if the *P*-value was <0.05.

## Results

### Baseline characteristics

A total of 178 patients who met the criteria were included in this study, including asthma (*n* = 38), ACO (*n* = 44), and COPD (*n* = 96). Statistical analysis showed significant differences in sex, age, smoking status, and pulmonary function between patients with asthma and patients with ACO and COPD, but there were no significant differences between ACO and COPD patients. Since the wide range of pulmonary function in patients with COPD, they were divided it into GOLD1-2 (*n* = 50) and GOLD3-4 (*n* = 46) for inter-group comparison in this study. The baseline characteristics of these patients are shown in Table 1. We found that there were more male patients, elderly people and smokers with ACO and COPD than with asthma, but there was no significant difference between ACO and COPD, which reflects the clinical characteristics of COPD (prevalent among smokers, the elderly and men). At the same time, it was found that the pulmonary function of ACO and COPD was worse than asthma, and ACO and GOLD3-4 were worse than GOLD1-2, while the difference between ACO and GOLD3-4 was not statistically significant. This shows that the airflow limitation is not serious or even completely normal in patients with stable asthma, while it is more serious in patients with ACO.

**TABLE 1:** Baseline characteristics.

Variable	Asthma	ACO	COPD		*P*-value
			GOLD1-2	GOLD3-4	
**Demographics**					
Sex, males (%)	17 (44.7)	32 (72.7)^#^	38 (76.0)^#^	38 (82.6)^#^	0.001
Age (years)	46.0 (14.50)	58.5 (13.00)^#^	62.0 (13.00)^#^	64.5 (12.25)^#^	< 0.001
BMI (kg/m^2^)	24.4 (4.49)	23.5 (5.22)	24.1 (3.21)	23.5 (5.07)	0.485
Smokers/ex-smokers (%)	8 (21.1)	25 (56.8)^#^	34 (68.0)^#^	34 (73.9)^#^	< 0.001
**Pulmonary function**					
FEV_1_/FVC (%)	69.5 (9.72)	51.2 (16.24)^#^*	64.2 (10.92)^#^	44.0 (19.40)^#^*	< 0.001
FEV_1_ (% pred)	83.3 (21.05)	42.9 (23.73)^#^*	66.3 (17.73)^#^	36.4 (17.25)^#^*	< 0.001
FEV_1_ (L)	2.6 (1.13)	1.4 (0.91)^#^*	1.9 (0.68)^#^	1.0 (0.53)^#^*	< 0.001
FVC (L)	3.6 (1.51)	2.8 (1.23)^#^	3.1 (1.11)	2.2 (0.98)^#^*	< 0.001

Enumeration data are presented as *n*, *n* (%), and measurement data are presented as median (IQR) unless otherwise stated. ACO, asthma-COPD overlap; COPD, chronic obstructive pulmonary disease; GOLD, Global Initiative for Chronic Obstructive Lung Disease; BMI, body mass index; FEV_1_, forced expiratory volume in 1 s; FVC, forced vital capacity. ^#^*P* < 0.05 vs. Asthma; *P < 0.05 vs. GOLD1-2.

### Differences in inflammatory index levels among asthma, asthma-COPD overlap, and chronic obstructive pulmonary disease

**FIGURE 1:**
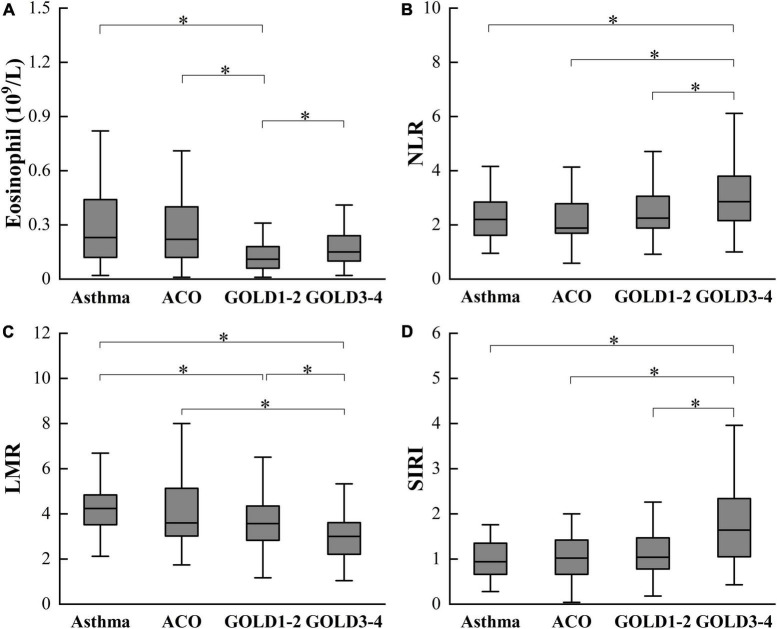
Differences in eosinophil **(A)**, NLR **(B)**, LMR **(C)**, and SIRI **(D)** among asthma, ACO and COPD. ACO, asthma-COPD overlap; COPD, chronic obstructive pulmonary disease; GOLD, Global Initiative for Chronic Obstructive Lung Disease; NLR, neutrophil-lymphocyte ratio; LMR, lymphocyte-monocyte ratio; SIRI, monocyte × neutrophil/lymphocyte. *P < 0.05.

There were statistically significant differences in eosinophil, NLR, lymphocyte-monocyte ratio (LMR), and SIRI between patients with asthma, ACO, and COPD (Table 2). Patients with COPD have lower eosinophil and LMR, higher NLR, and SIRI than patients with asthma and ACO. Moreover, eosinophil, NLR and SIRI in GOLD3-4 are higher than in GOLD1-2, while LMR is lower than in GOLD1-2. There was no significant difference in these inflammatory indexes between asthma and ACO. The differences between groups are shown in Figure 1.

**TABLE 2:** Inflammatory index levels among asthma, ACO, and COPD.

Inflammatory indexes	Asthma	ACO	COPD		*P*-value
			GOLD1-2	GOLD3-4	
Hemoglobin (g/L)	148.0 (24.00)	145.0 (29.50)	147.0 (20.50)	149.0 (27.75)	0.798
Platelet (10^9^/L)	235.0 (88.00)	213.0 (84.50)	215.0 (75.50)	203.5 (108.00)	0.376
Eosinophil (10^9^/L)	0.23 (0.320)	0.22 (0.290)	0.11 (0.125)	0.15 (0.150)	0.016
NLR	2.2 (1.22)	1.9 (1.21)	2.3 (1.26)	3.0 (1.62)	0.011
PLR	116.8 (57.00)	115.6 (69.87)	116.1 (34.12)	117.3 (80.63)	0.992
LMR	4.2 (1.32)	3.6 (2.15)	3.6 (1.62)	3.0 (1.37)	0.001
EBR	4.8 (9.07)	5.6 (4.88)	3.5 (4.15)	4.3 (3.96)	0.117
SII	522.2 (262.38)	412.1 (325.45)	445.4 (341.08)	554.5 (307.86)	0.192
SIRI	0.9 (0.69)	1.0 (0.86)	1.0 (0.77)	1.7 (1.27)	0.008

ACO, asthma-COPD overlap; COPD, chronic obstructive pulmonary disease; GOLD, Global Initiative for Chronic Obstructive Lung Disease; NLR, neutrophil-lymphocyte ratio; PLR, platelet-lymphocyte ratio; LMR, lymphocyte-monocyte ratio; EBR, eosinophil-basophil ratio; SII, platelet × neutrophil/lymphocyte; SIRI, monocyte × neutrophil/lymphocyte.

### To distinguish the inflammatory indexes of asthma, asthma-COPD overlap, and chronic obstructive pulmonary disease

This study used multinomial logistic regression to analyze the differences in inflammatory levels among patients with asthma, ACO, and COPD (Table 3). We found that with ACO as a reference, the higher the indexes of platelet, NLR, and eosinophil-basophil ratio (EBR), the more likely the patient will develop asthma, whereas the higher the indexes of eosinophil and platelet-lymphocyte ratio (PLR), the less likely the patient will develop asthma. In addition, compared to ACO, the higher the indexes of platelet, NLR, and EBR, the greater the possibility of COPD occurred, while the higher the index of eosinophil, the less likely COPD occurred.

**TABLE 3:** Multinomial logistic regression analysis for asthma, ACO, and COPD.

Predictors	Asthma OR (95% CI)	*P*-value	ACO	COPD OR (95% CI)	*P*-value
Hemoglobin (g/L)	0.99 (0.969–1.007)	0.197	–	1.00 (0.986–1.012)	0.846
Platelet (10^9^/L)	1.02 (1.003–1.034)	0.023	–	1.02 (1.002–1.029)	0.021
Eosinophil (10^9^/L)	0.03 (0.001–0.693)	0.029	–	0.001 (0–0.018)	<0.001
NLR	5.19 (1.384–19.483)	0.015	–	3.67 (1.176–11.45)	0.025
PLR	0.98 (0.964–0.995)	0.011	–	0.99 (0.976–1.002)	0.101
LMR	1.13 (0.798–1.602)	0.489	–	0.94 (0.692–1.265)	0.666
EBR	1.15 (1.023–1.295)	0.019	–	1.16 (1.031–1.298)	0.013
SII	1.00 (0.993–1.003)	0.418	–	1.00 (0.992–1.000)	0.076
SIRI	0.47 (0.132–1.702)	0.253	–	1.28 (0.441–3.737)	0.647

ACO, asthma-COPD overlap; COPD, chronic obstructive pulmonary disease; NLR, neutrophil-lymphocyte ratio; PLR, platelet-lymphocyte ratio; LMR, lymphocyte-monocyte ratio; EBR, eosinophil-basophil ratio; SII, platelet × neutrophil/lymphocyte; SIRI, monocyte × neutrophil/lymphocyte; OR, odds ratio; CI, confidence interval.

### Relationship between symptom and inflammatory indexes

Since the symptoms of asthma patients are not obvious in the stable period, we used the COPD assessment test (CAT) score to evaluate the symptoms of ACO and COPD patients. Through the correlation analysis of CAT score and inflammatory indexes, it was found that hemoglobin and LMR were negatively correlated with the severity of patients’ symptoms (Table 4). In other words, the lower the value of hemoglobin and LMR, the more serious the patient’s symptoms.

**TABLE 4:** Univariate and multivariate correlation between symptom severity and inflammatory indexes.

Predictors	Univariate		Multivariate	
	Coefficients	*P*-value	Coefficients	*P*-value
Sex	–0.082	0.114	0.069	0.611
Age	0.013	0.806	0.005	0.963
BMI	0.014	0.787	0.010	0.911
Smoking status	–0.197	0.020	–0.289	0.058
Hemoglobin	–0.229	< 0.001	–0.208	0.023
Platelet	0.024	0.651	–0.074	0.758
Eosinophil	0.142	0.007	0.192	0.152
NLR	0.170	0.001	–0.183	0.632
PLR	0.125	0.018	0.015	0.942
LMR	–0.302	< 0.001	–0.247	0.022
EBR	0.121	0.022	–0.046	0.730
SII	0.150	0.004	0.019	0.957
SIRI	0.198	< 0.001	0.265	0.383

BMI, body mass index; NLR, neutrophil-lymphocyte ratio; PLR, platelet-lymphocyte ratio; LMR, lymphocyte-monocyte ratio; EBR, eosinophil-basophil ratio; SII, platelet × neutrophil/lymphocyte; SIRI, monocyte × neutrophil/lymphocyte.

### Relationship between pulmonary function and inflammatory indexes

The relationship between inflammatory indexes and pulmonary function was analyzed by multiple linear regression (Table 5). We found that PLR was negatively correlated with FEV_1_/FVC and FEV_1%_ pred, respectively; age was negatively correlated with FEV_1_/FVC; body mass index (BMI) was positively correlated with FEV_1_/FVC; and EBR was positively correlated with FEV_1%_ pred.

**TABLE 5:** Multivariate correlation between pulmonary function and inflammatory indexes.

Predictors	FEV_1_/FVC		FEV_1%_ pred	
	Coefficients	*P*-value	Coefficients	*P*-value
Sex	–0.071	0.534	–0.063	0.585
Age	–0.201	0.034	–0.163	0.085
BMI	0.161	0.045	0.037	0.648
Smoking status	–0.046	0.724	–0.094	0.475
Hemoglobin	–0.067	0.404	0.028	0.728
Platelet	0.084	0.695	0.060	0.782
Eosinophil	–0.131	0.278	–0.201	0.098
NLR	0.229	0.495	0.315	0.350
PLR	–0.453	0.005	–0.407	0.012
LMR	0.116	0.241	0.191	0.055
EBR	0.162	0.164	0.257	0.029
SII	0.479	0.115	0.395	0.195
SIRI	–0.395	0.122	–0.403	0.116

BMI, body mass index; NLR, neutrophil-lymphocyte ratio; PLR, platelet-lymphocyte ratio; LMR, lymphocyte-monocyte ratio; EBR, eosinophil-basophil ratio; SII, platelet × neutrophil/lymphocyte; SIRI, monocyte × neutrophil/lymphocyte; FEV_1_, forced expiratory volume in 1 s; FVC, forced vital capacity; FEV_1%_ pred, FEV_1_ percent predicted.

## Discussion

Chronic respiratory disease is a heterogeneous group of disorders characterized by airway inflammation and airway obstruction, in which asthma and COPD are the most common disease entities (20). Although clinicians can easily recognize typical COPD and asthma, it is difficult to completely identify the undefined ACO, which affects the follow-up treatment of patients (6). Therefore, there is an urgent need for establishing biomarkers that can help distinguish CRD (8). Our study focuses on the comprehensive evaluation of easily accessible hemogram indexes, especially ratio indexes, which avoids the influence of individual differences and shows greater stability (16).

Our study analyzed the inflammatory indexes of asthma, ACO, and COPD, and found that the higher the indexes of platelet, NLR, and EBR, the greater the risk of asthma and COPD compared with ACO. That may be related to the pathological mechanism of the disease, including the inflammatory process and the increase of inflammation in the aggravation period. Some researchers have found that in addition to playing a critical role in hemostasis and thrombosis, platelets are also considered immune cells participated in various immune related processes (26). In patients with allergic asthma, platelet-specific derived mediators increased significantly in serum, indicating platelet activation (27). Meanwhile, platelets are recruited and located in the lung tissue, and participate in the pathogenesis and pulmonary inflammatory response of allergic asthma by interacting with dendritic cells, eosinophils, and neutrophils (28–30). Moreover, hypoxemia can promote platelet activation, and further activation occurs during acute exacerbation, which is a spiral process (16). Based on the above research, we also confirmed that PLR was negatively correlated with pulmonary function. In other words, platelet not only affect the occurrence and development of the disease, but also affect patients’ symptoms and pulmonary function.

The reason why high eosinophil is less likely the possibility of asthma and COPD compared with ACO, may be that the exacerbation frequency of ACO is higher than that of asthma or COPD alone (3, 31). A large number of studies have shown that eosinophil is closely related to the exacerbation of COPD and asthma (32–34). Compared with patients without eosinophilia, those with eosinophilia (≥300 cells/μl) had a higher frequency of readmission for acute exacerbation of COPD (AECOPD) during the 1-year follow-up period. And the incidence of AECOPD increased progressively with increasing blood eosinophil counts among stable patients with COPD (35). It was also found that high eosinophil count was an independent risk factor for two or more asthma exacerbations or any asthma emergency department visit or hospitalization (36). In a word, eosinophil is strongly associated with high-frequency exacerbation, while ACO exacerbations are more frequent than asthma or COPD alone. Therefore, high eosinophil is less likely to develop asthma and COPD compared with ACO. Because there are few studies on the inflammatory mechanism of ACO, we suspect that high platelet, NLR, and EBR are associated with the high risk of asthma and COPD with ACO as a reference, which may be related to the better response of ACO to pharmacological therapies, not least inhaled corticosteroids (4).

We also found that hemoglobin was negatively correlated with the severity of patients’ symptoms. Although it is traditionally considered that COPD is associated with polycythemia, a large number of studies have shown that anemia in COPD is more prevalent than expected (37). Anemia of chronic disease is a systemic inflammation driven by immunity, which is now recognized as a feature of COPD. Anemia in COPD may aggravate dyspnea and limit exercise tolerance, and increase the risk of mortality and exacerbation (38–40). Consequently, the lower the hemoglobin of patients, the more serious the symptoms.

In addition, our study confirmed that EBR was positively correlated with FEV_1_% pred. Although there is a line of evidence to show that eosinophil is related to exacerbation of patients (41), it is found that patients with persistent eosinophilia have significantly higher FEV_1_% pred and less dyspnea symptoms (42), which is consistent with our results. For this interesting phenomenon, the specific mechanism is still unclear and controversial. It is speculated that although eosinophil is related to exacerbations, patients with high eosinophil are sensitive to inhaled corticosteroid (ICS) (43), thus have a better response to therapy resulting in quicker recovery (13). Secondly, it was found that lower eosinophilic COPD patients had higher neutrophil counts. Neutrophilia is known to be a marker of bacterial infection, which is a common cause of exacerbations. The exacerbations of COPD caused by bacterial infection are associated with longer hospital stay (34). Although EBR is an eosinophil-related index, we believe that EBR tends to predict the prognosis of the disease, while eosinophil is related to the acute exacerbation of patients according to the results.

In summary, our study has established inflammatory biomarker to distinguish CRD by using easily accessible blood routine. These biomarkers can assist clinicians to accurately identify patients, thereby to maximize the effects of the personalized treatment (11). There are extremely differences in evidence-based treatment between asthma and COPD: because frequent use of ICS can increase the risk of pneumonia (44), it is recommended to use long-acting β_2_-agonist (LABA) and/or long-acting muscarinic antagonists (LAMA) alone (without ICS) as the initial treatment for COPD; but contraindicated for asthma and ACO due to the risk of serious deterioration and death (45). In addition, because the prognosis of patients with ACO is significantly worse, some guidelines recommend add-on therapy of LAMA to the basis treatment of ICS and LABA (i.e., triple therapy) (46). Therefore, whether we can accurately identify CRD is closely related to the follow-up personalized treatment (47).

Our study also has limitations. Firstly, this study was a single-center retrospective clinical study, which reflects the characteristics of specific populations and has regional limitations. Secondly, the number of samples in the study was limited. With the increase of the sample size, the sensitivity and specificity of the research results may also increase. At the same time, large samples can be divided into subgroups (according to acute exacerbation frequency, with/without ICS, et al.) to better understand CRD in many aspects. Thirdly, since there is no universally recognized definition of ACO currently, our diagnosis was based on the comprehensively assessment of clinical characteristics as well as the result of pulmonary function. Therefore, there may be some differences in the results of different diagnostic criteria. Finally, this study was designed as a cross-sectional study without long-term follow-up of patients, so it was difficult to evaluate the relationship between inflammatory indexes and patient outcomes. Despite these many limitations, we believe that our study shows that inflammatory indexes have clinical application value to a certain extent.

Although our research shows that inflammatory biomarkers can distinguish CRD, the clinical application value is still in the exploration stage. We mainly differentiate CRD according to diagnostic criteria and some clinical indicators that can reflect the characteristics of the disease (e.g., fractional exhaled nitric oxide, total immunoglobulin E, allergy tests, et al.) (48–50). Inflammatory biomarkers are just another auxiliary means for the hard-to-distinguish CRD.

## Conclusion

The results of this study show that patients with CRD can be distinguished by inflammatory indexes. In addition, it was also found that different inflammatory indexes have different effects. Platelet, NLR, and EBR can distinguish asthma, ACO, and COPD; hemoglobin and LMR were correlated with the severity of patients’ symptoms; PLR and EBR were related to pulmonary function. In clinical application, we can use different indexes to achieve different purposes, providing a new direction for accurate identification and individualized treatment in the future.

## Data availability statement

The raw data supporting the conclusions of this article will be made available by the authors, without undue reservation.

## Ethics statement

The studies involving human participants were reviewed and approved by the Medical Ethics Committee of Xijing Hospital of Air Force Medical University (No. KY20212201-C-1). The patients/participants provided their written informed consent to participate in this study.

## Author contributions

XT, SQ, and HM participated in the study design. LY, XZ, and XX tested the samples. LL, YZ, and XG contributed to the data collection. SW provided administrative support. HM conducted data analysis and wrote the manuscript. XT and SQ revised the manuscript. All authors have read and approved the final manuscript.
